# The thoracoacromial trunk: a detailed analysis

**DOI:** 10.1007/s00276-022-03016-4

**Published:** 2022-09-12

**Authors:** Michał Bonczar, Kamil Gabryszuk, Patryk Ostrowski, Jakub Batko, Daniel Jakub Rams, Agata Krawczyk-Ożóg, Wadim Wojciechowski, Jerzy Walocha, Mateusz Koziej

**Affiliations:** 1 Department of Anatomy, Jagiellonian University Medical College, Mikołaja Kopernika 12, 33-332 Kraków, Poland; 2 Chiroplastica-The Lower Silesian Center of Hand Surgery and Aesthetic Medicine, Wroclaw, Poland; 3 Department of Radiology, Jagiellonian University Medical College, Kraków, Poland

**Keywords:** Thoracoacromial trunk, Axillary artery, Subclavian artery, Perforator flaps

## Abstract

**Purpose:**

The thoracoacromial trunk (TAT) originates from the second part of the axillary artery and curls around the superomedial border of the pectoralis minor, subsequently piercing the costocoracoid membrane. Knowledge about the location, morphology, and variations of the TAT and its branches is of great surgical importance due to its frequent use in various reconstructive flaps.

**Methods:**

A retrospective study was conducted to establish anatomical variations, their prevalence, and morphometric data on TAT and its branches. The results of 55 consecutive patients who underwent neck and thoracic computed tomography angiography were analyzed. A qualitative evaluation of each TAT was performed.

**Results:**

A total of 15 morphologically different TAT variants were initially established. The median length of the TAT was set at 7.74 mm (LQ 3.50; HQ 13.65). The median maximum diameter of the TAT was established at 4.19 mm (LQ 3.86; HQ 4.90). The median TAT ostial area was set to 13.97 mm (LQ 11.70; HQ 18.86). To create a heat map of the most frequent location of the TAT, measurements of the relating structures were made.

**Conclusion:**

In this study, the morphology and variations of the branching pattern of the TAT were presented, proposing a new classification system based on the four most commonly prevalent types. The prevalence of each branch arising directly from the TAT was also analyzed. It is hoped that the results of the present anatomical analysis can help to minimize potential complications when performing plastic or reconstructive procedures associated with TAT.

## Introduction

The thoracoacromial trunk (TAT) originates from the second part of the axillary artery and curls around the superomedial border of the pectoralis minor, subsequently piercing the costocoracoid membrane. It then divides into four branches, the pectoral, deltoid, acromial, and clavicular branches [9].

Although rare, variations in the origin and branching pattern of TAT may occur, numerous examples have been presented in the literature, such as a case of an anomalous radial artery arising from the TAT [7]. The “norm” in anatomy is not as precise a concept as one would wish, and can be considered an approximation [19]. The branching pattern of the TAT has been a topic of great controversy, with some studies stating that the lateral thoracic artery predominately originates from the TAT rather than the axillary artery [8]. Additionally, other studies have reported that the acromial branch does not originate directly from the TAT but from other branches that do so [2, 4, 13].

Knowledge about the location, morphology, and variations of the TAT and its branches is of great surgical importance due to its frequent use in various reconstructive flaps. The TAT pedicled perforator flap has been used in tracheal reconstructive procedures. Several studies have described this type of flap as a good choice for the repair of tracheal defects due to its short harvest time, thin thickness, and stable blood supply [1]. Zhang et al. presented a cadaveric and clinical study on the potential clinical application of the TAT perforator flap [18]. The authors of the study found a constant TAT perforator in the septum of the clavicular and sternocostal heads of the pectoralis major muscle in 21 of 24 hemichests. Furthermore, they presented seven clinical cases in which the TAT flap was used, with no reported complications. Due to the importance of TAT in numerous reconstructive procedures, the goal of the present study was to provide surgically useful data on TAT and its relationship to surrounding structures. Furthermore, a heat map of the prevalence of the origin of TATs was presented. Different correlations and alliances between the analyzed parameters were also established. It is hoped that the results of this article can help minimize the potential surgical complications associated with TAT and its branches.

## Patients and methods

### Bioethical committee

The research protocol was submitted for evaluation and approved by the Bioethical Committee of Jagiellonian University, Cracow, Poland (1072.6120.51.2022). Further stages of the study were carried out according to the approved guidelines.

### Study group

A retrospective study was conducted to establish anatomical variations, their prevalence, and morphometric data on the TAT and its branches. The results of 55 consecutive patients who underwent neck and thoracic computed tomography angiography (CTA) were analyzed in the Department of Radiology of the Jagiellonian University Medical College, Cracow, Poland, in April 2022. The results of each patient were analyzed bilaterally. Therefore, a total of 110 TATs were initially evaluated. Exclusion criteria were established as follows: (1) neck and/or thoracic trauma affecting the course of the TAT and/or its initial branches, (2) significant artifacts that prevented accurate and precise imaging and/or measurement of the TAT and/or its initial branches, (3) low-quality and illegible images, and (4) significant lack of filling the whole arterial system with contrast. Defects that met the exclusion criteria but included only one side of the CT without interference with the contralateral side did not disqualify the entire CT, but only the affected side. Therefore, of the initial 110, a total of 61 TATs were excluded to minimize a potential bias. Of the 61, a great majority (*n* = 55) were excluded due to significant artifacts and/or lack of contrast filling. The rest (*n* = 6) were excluded due to being low-quality images. Eventually, 49 TATs met the required criteria.

### Results’ acquisition

All head and neck CTA were performed on a 128-slice scanner CT (Philips Ingenuity CT, Philips Healthcare). The main CT imaging parameters were as follows: collimation/increment: 0.625/0.3 mm; tube current: 120 mAs; field of view: 210 mm; matrix size: 512 × 512.

All patients received intravenous administration of contrast material at a dose of 1 ml/kg (standard dose). A non-ionic contrast medium (CM) containing 350 mg of iodine per mL was used (Jowersol 741 mg/mL, Optiray^®^, Guerbet, France). CT data acquisition was triggered using a real-time bolus-tracking technique (Philips Healthcare) with the region of interest (ROI) placed in the ascending aorta. The CM was intravenously injected using a power injector at a flow rate of 5 mL/s, which was immediately followed by injecting 40 mL of saline solution at the same flow rate. Following injection of CM and saline, image acquisition was automatically started with a 2 s delay when the attenuation trigger value reached a threshold of 120 Hounsfield units (HU). Scanning was performed in the caudocranial direction, while the CTA examination was started at the level of the aortic arch up to the circle of Willis.

The CTAs were analyzed on a dedicated workstation in the Anatomical Department of Jagiellonian University Medical College, Cracow, Poland. To ensure the highest possible quality of the visualizations and measurements and minimize potential bias, Materialise Mimics Medical version 22.0 software (Materialise NV, Leuven, Belgium) software was used. Three-dimensional (3D) reconstructions of each scan were developed, employing a set of settings, severally adjusted to each scan. Due to the nature of the contrast study, the cut-off level was set at the lower limit of normal, oscillating in the range of 25–80 HU. The range was individually adjusted to each TT after a visual investigation.

### Evaluation and measurements

**Fig. 1:**
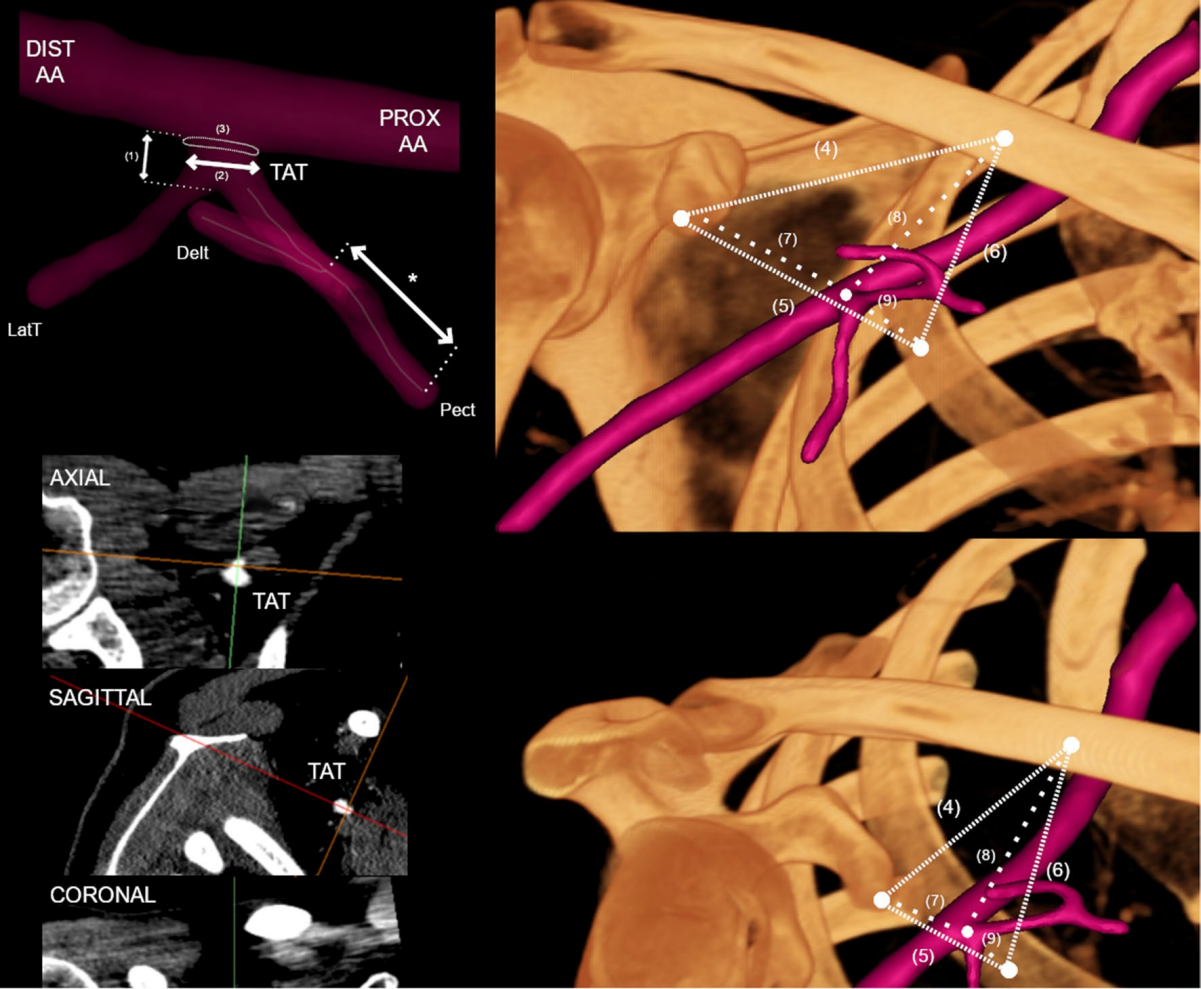
Measurement methodology presented in graphical form. To determine the TAT morphology, the multiplanar reconstruction of CT images was performed. *PROX AA* Proximal axillary artery; *DIST AA* distal axillary artery; *TAT* thoracoacromial trunk; *Delt* deltoid branch of TAT; *LatT* lateral thoracic artery; *Pect* pectoral branch of TAT; (1) Length of TAT; (2) maximal diameter of TAT; (3) TATs’ ostial area; (4) distance from the coracoid process to the clavicle, (5) distance from the coracoid process to the second rib, (6) distance from the clavicle process to the second rib, (7) distance from the coracoid process to the TAT, (8) distance from the clavicle to the TAT, and (9) distance from the second rib to the TAT. An asterisk (*) indicates the length of the corresponding consecutive Pect of TAT or its branches

Each TAT, including its course, branches, and close anatomical area, was fully visualized at the beginning of each evaluation. Furthermore, each branch of the TAT was identified by following its course. The direction of the TAT and a set of its branches with their arrangement were evaluated and descriptively noted. Afterward, a set of measurements was executed on each TAT. The literature lacks a clear definition of the TAT and how its length should be measured; therefore, the authors decided that the TAT length would be established as the shortest distance followed over the surface of the TAT (from the axillary artery to the point of the first branch). The authors recognize the TAT as an arterial branch of the axillary artery, which ends at the sprouting point of its first branch. On each TAT, a set of parameters were measured: (1) TATs length, (2) TATs maximal diameter at its origin, (3) TATs ostial area at its origin, (4) distance from the coracoid process to the clavicle, (5) distance from the coracoid process to the second rib, (6) distance from the clavicle process to the second rib, (7) distance from the coracoid process to the TAT, (8) distance from the clavicle to the TAT, and (9) distance from the second rib to the TAT. Additionally, each pectoral branch has been measured. To maintain objectivity, the same measurement pattern was followed when establishing distances between structures. Additionally, each trunk was qualitatively evaluated with respect to its branches, their pattern, and their subsequent branches. Each measurement was obtained twice, by two independent researchers, and a mean value was established afterward. The measurement methods used in this study are presented in Fig. 1.

### Statistical analysis

Statistical analysis was performed with SATISTICA v13.1 (StatSoft Inc., Tulsa, OK, USA). The frequency and percentages presented qualitative features. The Shapiro–Wilk test was used to assess the normal distribution. Quantitative characteristics were presented by medians and upper and lower quartiles (UQ, LQ), as well as means and standard deviation (SD), depending on the verified normality of the data. Statistical significance was defined as *p* < 0.05. *U* Mann–Whitney and Wilcoxon signed-rank tests were used to establish potential differences between groups. Spearman’s rank correlation coefficient was used to determine possible correlations between the parameters.

## Results

### Baseline characteristics

A total of 49 TATs were from patients between 15 and 82 years of age, with a mean age of 50.57 (SD 18.39), of which 21 (42.86%) were from women and 28 (57.14%) were from men. The left sides were analyzed in 24 cases (48.98%) and the right sides in 25 cases (51.02%). The direction of each TAT was evaluated. The trunk originated more frequently in the superior position (81.63%), but in some cases, it originated anteriorly (18.37%).

### TAT variations

**Fig. 2:**
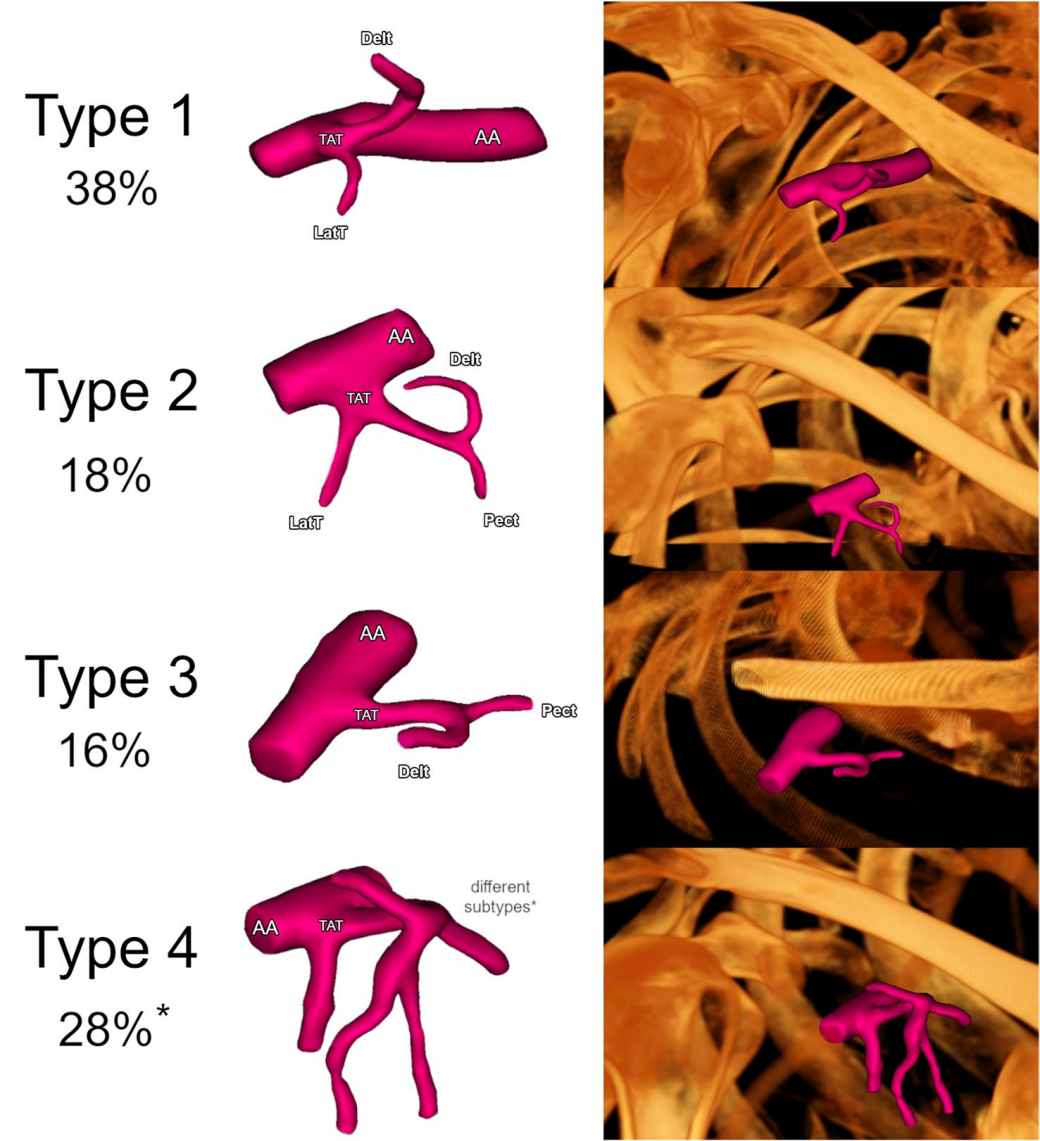
Summary of the most common morphological types of TAT. Type 1: TAT arises from the second part of the axillary artery. First, the lateral thoracic artery branches out, and then the deltoid artery. Type 2: TAT arises from the second part of the axillary artery. The lateral thoracic, pectoral, and deltoid arteries branch out sequentially. Type 3: TAT arises from the second part of the axillary artery. First, the pectoral artery branches out, and then, the deltoid artery branches off. Type 4: Any other variation. An asterisk (*) indicate Type 4—Other, which is not an individual type but a separate set for the other TAT morphological types that do not fit Types 1–3

A qualitative evaluation of each TAT was performed. Five different arteries branching off directly from the TAT were noted: (1) pectoral artery; (2) acromial artery; (3) clavicular artery; (4) deltoidal artery, and (5) lateral thoracic artery. Repetitively, in all the evaluated cases, the pectoral arteries ran anterolaterally toward the walls of the thoracic cage, the clavicular arteries ran to the clavicles, the deltoid arteries ran to the deltoideus muscles, and the acromial arteries ran to the muscles in the acromial region. No exceptions were noted. In general, a total of 15 morphologically different TAT variants were established. However, only three of them showed significant repeatability. Therefore, a classification system was developed for branches that originate directly from the TAT and 4 main types were set as follows: (1) Type 1: TAT arises from the second part of the axillary artery. First, the lateral thoracic artery branches out, and then the deltoid artery. (2) Type 2: TAT arises from the second part of the axillary artery. The lateral thoracic, pectoral, and deltoid arteries branch out sequentially. (3) Type 3: TAT arises from the second part of the axillary artery. First, the pectoral artery branches out and then the deltoid artery branches out. (4) Type 4: Any other variation. Type 1 was found in 19 of the cases (38.0%), type 2 was found in 9 of the cases (18.0%), and type 3 was found in 8 cases (16.0%). Type 4 consisted of the 11 remaining types evaluated. However, none of these types was observed more than two times. The other variations, which were represented as type 4, were observed in 14 of the cases (28.0%). Types 1, 2, and 3 are illustrated in Fig. 2. Taking into account branches that originate directly from TAT, the prevalence of each recognized artery has been demonstrated. The lateral thoracic artery branched directly out of TAT in 31 (63.3%) of the cases. Furthermore, the pectoral artery branched out in 29 (59.2%), the clavicular artery in 8 (16.3%), the acromial artery in 2 (4.1%), and the deltoid artery in 46 (93.9%) of the cases.

### General measurements

**Fig. 3:**
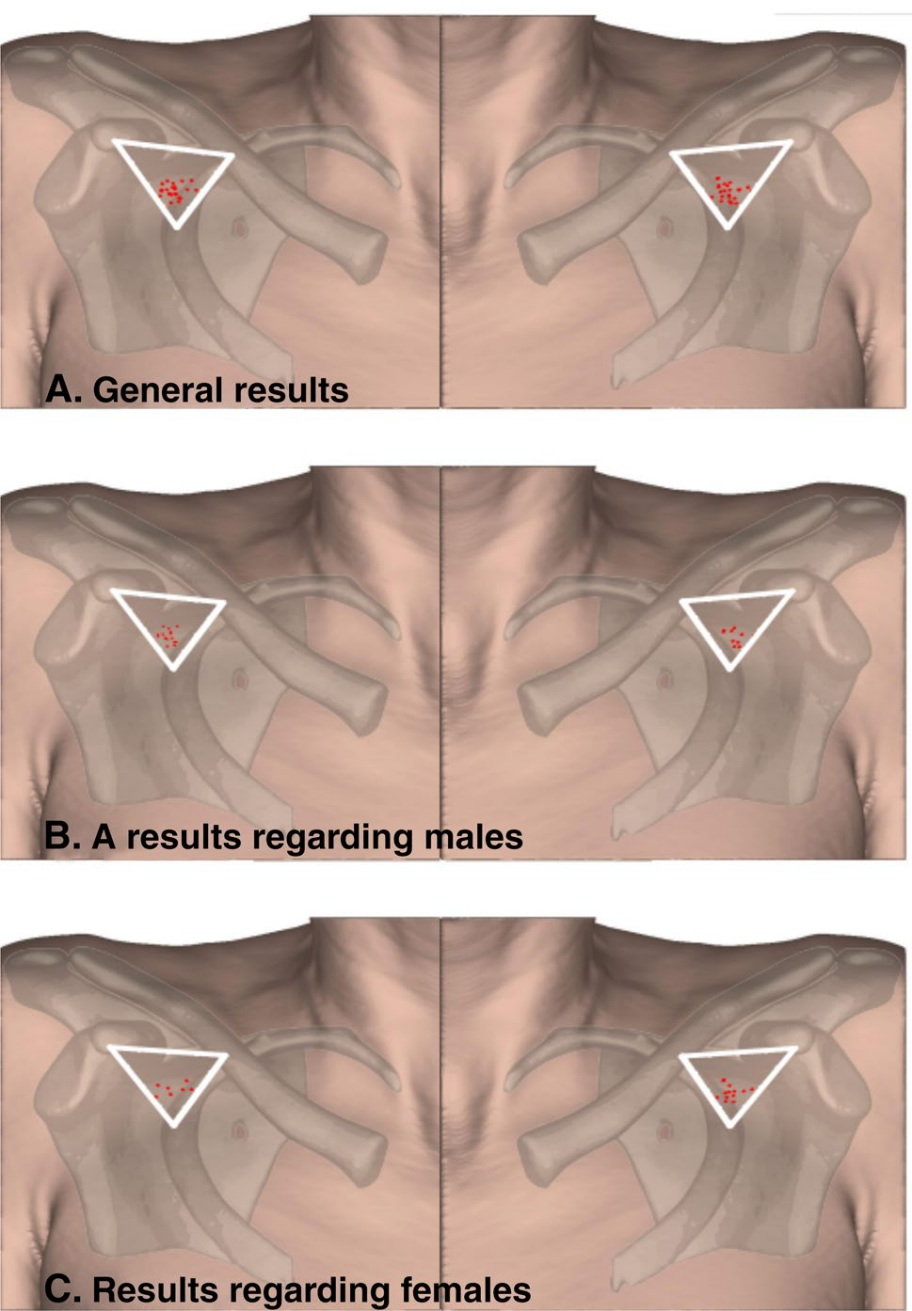
An anatomical map of the occurrence of TAT in the clavipectoral triangle. **A** general results. **B** results regarding males. **C** results regarding females

The median length of the TAT was set at 7.74 mm (LQ 3.50; HQ 13.65). The median maximum diameter of the TAT was established at 4.19 mm (LQ 3.86; HQ 4.90). The median TAT ostial area was set to 13.97 mm (LQ 11.70; HQ 18.86). To create a heat map of the most frequent location of the TAT, measurements of the relating structures were made. The median distance from the coracoid process to the clavicle was established at 48.42 mm (LQ 40.88; HQ 53.12). The median distance from the coracoid process to the second rib was established at 50.88 mm (LQ 40.17; HQ 56.13). The median distance from the clavicle to the second rib was established at 45.29 mm (LQ 38.32; HQ 49.73). The median shortest distance from the coracoid process to TAT was established at 33.55 mm (LQ 28.20; HQ 40.87). The median shortest distance from the clavicle to the TAT was established at 30.99 mm (LQ 27.66; HQ 36.63). The median shortest distance from the second rib to the TAT was set to 18.18 mm (LQ 14.31; 22.82). The aforementioned results allowed the authors to create an anatomical map of the occurrence of TAT in the clavipectoral triangle. The anatomical map is illustrated in Fig. 3. The length of the pectoral artery was also determined due to its clinical significance in reconstructive flaps. Furthermore, its origin (whether it originates directly or indirectly from TAT) and abundance have been taken into account. The median length of the pectoral branch, branching directly off from the TAT was set to be 70.54 [mm] (LQ 68.72; HQ 75.54). All the results mentioned above and more detailed descriptions are presented in Table 1.

**Table 1:** General results of the measurements

Category	Median	LQ	HQ	Minimum	Maximum	Mean	SD
TAT length [mm]	7.74	3.50	13.65	0.86	22.42	9.23	6.09
TAT maximal diameter [mm]	4.19	3.86	4.90	2.24	5.53	4.27	0.67
TAT ostial area [mm]	13.79	11.70	18.86	3.94	24.02	14.68	4.40
Distance from the coracoid process to the clavicle [mm]	48.42	40.88	53.12	28.99	63.06	47.02	8.48
Distance from the coracoid process to the second rib [mm]	50.88	40.17	56.33	30.73	67.72	49.28	9.78
Distance from the clavicle to the second rib [mm]	45.29	38.32	49.73	27.09	64.40	44.45	8.31
Distance from the coracoid process to the TAT [mm]	33.55	28.20	40.87	18.06	67.38	35.19	9.32
Distance from the clavicle to the TAT [mm]	30.99	27.66	36.62	17.67	49.53	31.74	6.95
Distance from the second rib to the TAT [mm]	18.18	14.31	22.82	7.29	29.77	18.39	5.95
Length of the first pectoral branch. branching off from TAT [mm]	70.54	68.72	75.64	62.25	82.34	71.68	5.21
Length of the second pectoral branch. branching off from TAT [mm]	63.57	59.62	65.99	51.35	78.75	63.81	9.08
Length of the first pectoral branch. branching off from any of the TATs branches [mm]	33.25	29.21	36.92	21.31	50.32	33.17	6.37
Length of the second pectoral branch. branching off from any of the TATs branches [mm]	29.89	27.42	31.30	22.36	34.50	29.09	4.55

*LQ* lower quartile, *HQ* higher quartile, *SD* standard deviation, *TAT* thoracoacromial trunk

### Sexual dimorphism

Regarding all the categories mentioned in Table 1, the potential differences between men and women were analyzed. The measurement results obtained from male cases were significantly higher in 7 of 9 categories (*p* < 0.05): (1) TAT maximal diameter; (2) TAT ostial area; (3) distance from the coracoid process to the clavicle; (4) distance from the coracoid process to the second rib; (5) distance from the clavicle to the second rib; (6) distance from the coracoid process to the TAT; (7) distance from the second rib to the TAT. All the results mentioned above and more detailed descriptions of sex differences are presented in Table 2.

**Table 2:** Results of measurements with respect to sex

Category	Sex	Median	LQ	HQ	Minimum	Maximum	Mean	SD	*P *value
TAT length [mm]	Female	7.14	3.41	10.80	0.86	18.10	7.53	5.01	0.10
	Male	10.42	4.27	16.97	2.10	22.42	10.51	6.59	
TAT maximal diameter [mm]	Female	3.86	3.50	4.48	2.24	5.31	4.00	0.73	0.01
	Male	4.53	4.06	4.95	3.31	5.53	4.48	0.56	
TAT ostial area [mm]	Female	11.70	9.62	15.76	3.94	22.14	12.98	4.54	0.01
	Male	16.08	12.92	19.21	8.60	24.02	15.96	3.91	
Distance from the coracoid process to the clavicle [mm]	Female	41.78	35.07	48.18	28.99	56.77	42.19	7.92	0.00
	Male	50.23	46.40	54.32	34.82	63.06	50.61	7.08	
Distance from the coracoid process to the second rib [mm]	Female	42.57	38.23	52.14	30.73	61.48	44.28	9.45	0.00
	Male	54.03	48.21	58.28	33.56	67.72	52.99	8.39	
Distance from the clavicle to the second rib [mm]	Female	39.13	37.10	44.05	27.09	52.33	40.33	6.52	0.01
	Male	47.45	44.54	53.96	29.09	64.40	47.55	8.24	
Distance from the coracoid process to the TAT [mm]	Female	30.50	25.73	36.55	18.06	67.38	32.61	11.00	0.02
	Male	37.10	30.22	42.26	26.80	54.53	37.11	7.51	
Distance from the clavicle to the TAT [mm]	Female	28.49	23.41	31.24	17.67	42.79	28.53	6.74	0.06
	Male	34.87	29.93	37.98	21.53	49.53	34.15	6.18	
Distance from the second rib to the TAT [mm]	Female	15.34	13.96	17.15	8.60	29.77	16.82	5.50	0.01
	Male	19.94	15.32	23.91	7.29	28.79	19.57	6.09	
Length of the first pectoral branch. branching off from TAT [mm]	Female	71.78	69.71	65.59	82.34	67.20	75.64	5.18	0.77
	Male	71.78	69.71	65.59	82.34	67.20	75.64	5.18	
Length of the first pectoral branch. branching off from any of the TATs branches [mm]	Female	32.72	33.65	21.31	40.17	28.35	37.23	5.80	0.94
	Male	32.72	33.65	21.31	40.17	28.35	37.23	5.80	
Length of the second pectoral branch. branching off from any of the TATs branches [mm]	Female	29.36	29.36	27.42	31.30	27.42	31.30	2.74	1.00
	Male	29.36	29.36	27.42	31.30	27.42	31.30	2.74	

*LQ* lower quartile, *HQ* higher quartile, *SD* standard deviation, *TAT* thoracoacromial trunk

### Associations between parameters

Potential correlations were analyzed for each of the categories and for the age. The only parameter that statistically correlated with age was the distance from the coracoid process to the TAT (*R* = −0.32; *p* = 0.02). However, some categories correlate with each other. The associations between the categories are summarized in Table 3.

**Table 3:** Table gathers the R values obtained in the correlation analysis between categories

Category	Age [mm]	TAT length [mm]	TAT maximal diameter [mm]	TAT ostial area [mm]	Distance from the coracoid process to the clavicle [mm]	Distance from the coracoid process to the second rib [mm]	Distance from the clavicle to the second rib [mm]	Distance from the coracoid process to the TAT [mm]	Distance from the clavicle to the TAT [mm]	Distance from the second rib to the TAT [mm]
Age [mm]	1.00	0.09	−0.04	−0.04	−0.11	−0.03	0.01	*−0.32*	−0.16	0.28
TAT length [mm]	0.09	1.00	0.09	0.09	0.13	0.27	*0.31*	0.18	−0.01	*0.37*
TAT maximal diameter [mm]	−0.04	0.09	1.00	1.00	0.26	0.18	0.18	0.20	0.05	0.16
TAT ostial area [mm]	−0.04	0.09	1.00	1.00	0.26	0.18	0.18	0.20	0.05	0.16
Distance from the coracoid process to the clavicle [mm]	−0.11	0.13	0.26	0.26	1.00	*0.39*	0.27	*0.54*	*0.61*	−0.05
Distance from the coracoid process to the second rib [mm]	−0.03	0.27	0.18	0.18	*0.39*	1.00	*0.62*	*0.77*	0.21	*0.62*
Distance from the clavicle to the second rib [mm]	0.01	*0.31*	0.18	0.18	0.27	*0.62*	1.00	*0.29*	*0.55*	*0.79*
Distance from the coracoid process to the TAT [mm]	*−0.32*	0.18	0.20	0.20	*0.54*	*0.77*	*0.29*	1.00	0.12	0.20
Distance from the clavicle to the TAT [mm]	−0.16	−0.01	0.05	0.05	*0.61*	0.21	*0.55*	0.12	1.00	0.12
Distance from the second rib to the TAT [mm]	0.28	*0.37*	0.16	0.16	−0.05	*0.62*	*0.79*	0.20	0.12	1.00

Italics values are those in which the *p* value was smaller than 0.05

## Discussion

The current study proposed a classification system for the branches that originated directly from the TAT. However, in many cases, these branches do not originate directly from the said stem, but rather form common trunks or originate from each other. If the classification of the branch pattern of the TAT consisted of additional branches that originated from the arteries that directly arose from the TAT, the number of branch types would simply be too high. This would add unnecessary confusion with respect to the morphology and variations of this structure, contradicting the objective of this study. Therefore, the classification system consisted of only direct branches of the TAT. The prevalence of each artery which directly arose from the TAT was analyzed, showing that the pectoral (59.2%), the deltoid (93.9%), and the lateral thoracic arteries (63.3%) were the most frequent branches of the TAT. Similar results considering the origin of the pectoral artery were presented by Park et al. [11]. Surprisingly, the clavicular (16.3%) and acromial branches (4.1%) were less prevalent, demonstrating how variable the branching of the TAT is. In some cases, the arteries that were supposed to come out of the TAT originated from smaller arterial trunks or directly from the axillary artery. This phenomenon was observed more frequently in the clavicular and acromial arteries compared to the other branches of the TAT. Interestingly, TAT has been reported to have only three main branches, namely, the pectoral, clavicular, and deltoid arteries [2]. Studies have also described the acromial artery as almost always originating from the deltoid artery, rather than directly from the TAT [2, 4, 13]. The present study shows that the acromial artery is the least common branch that originates directly from the TAT, which is consistent with the description aforementioned. Furthermore, the clavicular branch was found to be a relatively rare (16.3%) direct branch of the TAT. There are different descriptions about the clavicular artery in the literature. Hallock et al. described the clavicular branch as variable and having a different origin than from the TAT [4]. However, Geddes et al. stated that the TAT gives rise to three major branches, with the clavicular artery being one of them [2]. Therefore, the authors of the present study would like to propose a new description of the direct branches of the TAT. The trunk should be described as an origin point for three arteries: the pectoral, the lateral thoracic, and the deltoid arteries. Furthermore, the acromial artery arises most frequently from the deltoid branch, whereas the clavicular artery has a highly variable origin and may even arise from the axillary artery.

The lateral thoracic artery was shown to originate more commonly from the TAT (59.2%) than the second part of the axillary artery, as described in the anatomical textbooks. Studies that present similar results have been published in the literature. Loukas et al. conducted a cadaveric study, consisting of 420 subjects fixed to formalin, where variations of the lateral thoracic artery were analyzed [8]. The study showed that the artery arises more frequently from the TAT (67.62%) and not directly from the axillary artery. This branching pattern of the TAT was also presented by other studies with lower frequencies, such as the study conducted by Pandey and Shukla [10]. In the study, the prevalence of the lateral thoracic artery arising from the TAT was reported separately on the left and right sides with an occurrence of 21.3% and 39.9%, respectively. These results indicate that the origin point of the lateral thoracic artery should be reconsidered.

Knowledge of the location, morphology, and variations of the TAT and its branches may be of great importance in plastic and reconstructive surgeries. Zhang et al. presented an extensive study providing evidence of the vascular supply and the clinical application of the perforator flap of the thoracoacromial artery [18]. In the study, both cadaveric and clinical investigations of the TAT were carried out. First, 12 cadavers were dissected to define the anatomy of the branches of the TAT and their perforators and the anatomical landmarks for clinical applications. They confirmed the presence of a constant perforator from the pectoral branch of the TAT, which allowed the design of a standard thoracoacromial artery perforator flap with a mean pedicle length of 7.1 cm. In the current study, the mean length of the pectoral branch was measured to be 7.0 cm (70.54 mm), which is very similar to the results presented by the aforementioned study, strengthening the data on pedicle length. Furthermore, Zhang et al. presented seven clinical cases in which the thoracoacromial artery perforator flap was used to reconstruct head and neck defects, with no reported complications. However, Geddes et al. reported results that were in contrast to those in the study conducted by Zhang et al. [2]. Similarly, a cadaveric study was performed to analyze the anatomy of the TAT, its branches, and the perforators. The average diameter of the TAT at the axillary artery was measured to be 2.5 ± 0.5 mm, which is a considerably lower value than the one reported in the present study (4.19 ± 0.67 mm). Furthermore, they concluded that due to the inconsistent size of the pectoral branch of the TAT and the small size of the musculocutaneous perforators, the pectoral branch of the TAT is not a good donor site for pedicled perforator flaps. However, they also stated that the musculocutaneous perforator flaps from the clavicular and deltoid branches of the TAT were possible. However, multiple studies have shown that the pectoral branch is a reliable pedicle for reconstructive flaps [4, 14, 16, 18]. When performing the thoracoacromial perforator flap or any other reconstructive flap pedicled by the TAT, knowing the location of the TAT is of great value. Therefore, a heat map of the occurrence of TAT in the clavipectoral region was made by measuring the relations of the trunks with the coracoid process and the second rib. This can help the surgeon create a mental map of the origin of the vessels, thus decreasing the risk of potential surgical complications when performing reconstructive procedures associated with the TAT.

The TAT can be used as a pedicle for flaps with various reconstructive applications. Pectoralis major muscle flaps are used to fill sternal defects and the conventional turnover method of this flap requires the internal thoracic vessels [6]. However, this method cannot be used when these vessels are sacrificed. Therefore, Goishi et al. developed a new major pectoralis turnover flap based on TAT vessels [3], which has proven to be a reliable technique for the reconstruction of long sternal defects. The TAT can also be used as a pedicle for sequential perforator flaps, which can repair the donor site of sternocleidomastoid myocutaneous flaps [15]. The acromial branch of the TAT can be used in a local perforator flap, for covering shoulder defects which is useful in clinical practice for reconstruction and covering of the acromial area with a thin cutaneous flap with low sequelae on the donor site [12]. Additionally, a free TAT true-perforator flap can be a new option for the reconstruction of the face with many advantages including reduced donor-site morbidity and satisfactory aesthetic outcome [5]. The TAT can be also safely used as recipient vessel for deep inferior epigastric artery perforator flap breast reconstruction with shorter time and less postoperative pain [17].

The present study undoubtedly has some limitations. Although the size of the study group used in the current paper is the largest among imaging studies concerning the TAT, larger population-based research is still warranted to discern the true prevalence of its variants. Additionally, radiological imaging only allows one to evaluate hemodynamically efficient arteries. Therefore, this can be a relatively significant source of bias when assessing anatomical variations of the TAT, and other arterial entities. Unfortunately, the morphometric properties of the perforators of the TAT could not be analyzed because of the insufficient resolution of the CTAs. Therefore, this topic requires further investigation, preferably by conducting a cadaver study. To get a better understanding of the general anatomy of the TAT, a detailed meta-analysis should be performed with respect to the guidelines and with the usage of tools that minimize potential bias [16].

## Conclusion

In this study, the morphology and variations of the branching pattern of the TAT were presented, proposing a new classification system based on the four most commonly prevalent types. The prevalence of each branch arising directly from the TAT was also analyzed. The most prevalent branches were the pectoral (59.2%), the deltoid (93.9%), and the lateral thoracic (63.3%) arteries. Interestingly, the acromial artery was the least common direct branch of the TAT, and the clavicular artery showed a high degree of variability in its origin. It is hoped that the results of the present anatomical analysis can help to minimize potential complications when performing plastic or reconstructive procedures associated with TAT.

## Data Availability

Data are not available due to ethical restrictions.
